# Oximetry-supported self-management for chronic obstructive pulmonary disease: mixed method feasibility pilot project

**DOI:** 10.1186/s12913-015-1135-2

**Published:** 2015-10-26

**Authors:** Michele MacNab, Siew Hwa Lee, Lucy McCloughan, Janet Hanley, Brian McKinstry, Hilary Pinnock

**Affiliations:** e-Health Research Group, Usher Institute of Population Health Sciences and Informatics, The University of Edinburgh, Edinburgh, UK; Department of Nursing and Midwifery, Faculty of Education and Health Sciences, University of Limerick, Limerick, Ireland; School of Nursing, Midwifery and Social Care, Edinburgh Napier University, Edinburgh, UK; Allergy and Respiratory Research Group, Usher Institute of Population Health Sciences and Informatics, The University of Edinburgh, Doorway 3, Medical School, Teviot Place, Edinburgh, EH8 9AG UK

**Keywords:** Chronic obstructive pulmonary disease (COPD), Self-management, Telemonitoring, Primary care, Delivery of care

## Abstract

**Background:**

Pulse oximetry could potentially contribute to self-monitoring. NHS Lothian’s ‘Light Touch’ service provided COPD patients with a self-management plan based on symptoms and oximetry. The service was overseen (though not actively monitored) by respiratory-trained community teams who were contactable by a telephone helpline. We aimed to assess the feasibility, perceived utility and impact of the ‘Light Touch’ service.

**Methods:**

A before-and-after assessment of St George’s Respiratory Questionnaire (SGRQ), Hospital Anxiety and Depression Scale (HADS) and use of healthcare resources during the 6-month feasibility study compared to the previous corresponding 6-months. Paired semi-structured interviews with patients at baseline and 6-months, interviews with managers and a focus group of professionals explored perceptions of the service and self-management. Transcripts were coded, and analysed thematically.

**Results:**

We recruited 51 participants (mean age 69.7 years (SD 8.4); 21 (46 %) male). 46 participants completed quantitative follow up (2 died, 2 were unwell, 1 refused). SGRQ: 21 (46 %) participants improved by 4 or more (the minimum important difference); 12 (26 %) deteriorated by 4 or more. HADS: more participants had normal scores for anxiety (65 %) and depression (80 %) at 6-months than at baseline (51 and 64 %). More emergency therapy was prescribed during the study period compared to the previous year. Only 18 participants (39 %) contacted the Light Touch Helpline during the 6-month study.

Twenty patients provided a total of 36 interviews, 8 clinicians contributed to a focus group and 6 managers were interviewed. Patients considered that the oximetry readings heightened awareness of their condition and gave them confidence to make self-management decisions. Healthcare professionals valued oximetry as a tool for teaching people self-management skills, but were concerned that patients rarely contacted the teams for help or advice during the study.

**Conclusions:**

‘Light Touch’ shows promise as a low-cost strategy for empowering patients’ self-management skills and reducing reliance on clinical supervision.

**Electronic supplementary material:**

The online version of this article (doi:10.1186/s12913-015-1135-2) contains supplementary material, which is available to authorized users.

## Background

Chronic Obstructive Pulmonary Disease (COPD) is a major cause of death, disability and hospital admissions due to exacerbations [1, 2]. Enabling people to monitor their condition, detect early symptoms of exacerbations [3], self-manage and/or seek timely advice for exacerbations may reduce admissions and improve health-related quality of life [4].

Health services globally promote telemonitoring to support self-management of long-term conditions, including COPD [5–7]. Typically, symptoms and physiological measures recorded by patients at home are transmitted to a web-based interface monitored by a professional [8–10]. If monitoring data breach pre-determined thresholds, the clinician is alerted and contacts the patient to discuss treatment options [11, 12].

However, in our recent trial, telemonitoring of COPD did not reduce hospital admissions, improve health-related quality-of-life or provide a more cost effective method of supporting patients with COPD [13, 14]. A perception that routine clinician monitoring may sometimes engender dependence on professional support and may not be necessary as patients became adept at recognising and responding to their own pattern of symptoms, [15] encouraged NHS Lothian to develop a novel ‘Light Touch’ approach in which patients recorded symptoms and oximetry but these were not monitored. Patients were responsible for contacting professionals if their condition deteriorated.

Positioned in the ‘refining’ phase of the development and evaluation of complex interventions, [16] this feasibility study used mixed methods to explore patients’ and professionals’ experience of the Light Touch service, in order to inform service development and identify potential outcome measures for future evaluation.

## Methods

The study, funded by the Edinburgh and Lothians Health Foundation, ran from 2013 to 2014, with National Research Ethics Service East Midlands – Leicester Committee approval (ref:12/EM/0359) and NHS Lothian Governance Approval (ref:2012/P/GP/06).

### Participant recruitment

People with symptomatic COPD (Medical Research Council Dyspnoea score ≥3 [17]) and at risk of admission due to a COPD exacerbation were identified and clinical eligibility assessed for the Light Touch service by professionals from community-based respiratory or long-term condition teams. The community team initiated the Light Touch monitoring, told patients about the study, gave brief written information, and requested permission to pass contact details to the research team. The researcher sent an information leaflet to potential participants, followed up with a phone call to assess their willingness to participate. Written consent was obtained during a home visit and baseline data collected (MM).

### Intervention

**Fig. 1:**
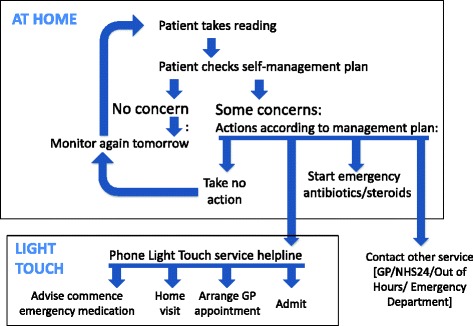
Light Touch intervention

Figure 1 illustrates the Light Touch intervention Participants recorded symptoms and pulse oximetry in a daily diary (Additional file 1). Community-based professionals visited patients at home, demonstrated the pulse oximeter, and provided the self-management plan which explained the symptoms and/or physiological measures which should trigger emergency self-treatment and/or contact with the Light-Touch telephone helpline (Additional file 2). Follow-up telephone calls or visits ensured they understood the process.

### Quantitative evaluation

#### Data collection

At baseline and six months after recruitment, participants completed the St George’s Respiratory Questionnaire (SGRQ) which assesses perception of respiratory problems, [18] Hospital Anxiety and Depression Scale (HADS) which assesses anxiety and depression, [19] EuroQol-5D(EQ-5D) a generic measure of health-status, [20] and Patient Activation Measure (PAM) which measures activation in self-management of chronic illness [21, 22].

Use of healthcare resources (including Light-Touch helpline use, COPD-related general practice consultations, accident and emergency (A&E) visits, outpatient appointments and hospital admissions, respiratory drugs) was collected from GP and community team records for the six-month duration of the study and for the corresponding six months of the previous year. Data were entered into the study database (MM), with data entry from a random 10 % of participants duplicated by a researcher not associated with the project in order to check for accuracy. No significant errors were detected.

#### Sample size and data analysis

We did not perform a sample size calculation for this pilot study. We aimed to recruit fifty patients as being sufficient for assessment of feasibility and to inform power calculations for a future trial. To prevent over interpretation of the data from this small pilot dataset we are not presenting statistical analyses and the majority of quantitative data is represented graphically.

### Qualitative evaluation

#### Data collection

We aimed to purposively recruit up to 20 Light-Touch participants representing maximum diversity of demographic and clinical characteristics expected to impact on views and attitudes to the service. Paired face-to-face semi-structured interviews shortly after recruitment and after six-months enabled the evolution of perceptions to be explored. Interviews were conducted by SHL, a qualitative researcher who was not involved in clinical service, in the patients’ homes with carers if the patient wished. Initial interviews lasted 40–60 min: follow-up interviews were approximately 20–30 min. To enhance rigour, the topic guide (Additional file 3) which explored the experience of Light-Touch and specifically if/how the daily readings contributed to managing their COPD, was informed by emerging themes and refined iteratively in discussion with the study team (SHL, HP, JH, MM, LMcC, BMcK).

An hour-long focus group, facilitated by SHL and LMcC, was carried out with professionals from the community teams to explore experiences and perspectives on the Light-Touch service. Face-to-face semi-structured interviews lasting between 30 and 40 min with the service managers (SHL) explored experiences and perceptions of the service, barriers/facilitators to implementation and future direction of the service.

Interviews and focus group were audio-recorded. With the patient’s consent at 6 months we took a digital photograph of their diary and self-management plan.

### Analysis

Interviews and focus group were transcribed and imported into NVivo v10 (QSR International, Melbourne) for organisation and facilitation of data analysis. Data were read and coded (by SHL) and analysed thematically using the ‘framework’ approach [23]. The codes and emerging themes were developed in detailed and frequent discussion with two senior researchers (JH: a qualitative researcher, HP: an academic general practitioner) to enhance rigour. Thematic analysis focused on patients’, healthcare professionals’ and managers’ experience of the Light-Touch service and specifically how this contributed (or not) to day-to-day management of COPD (Additional file 4 gives the coding framework).

The photographs of the patients’ monitoring records were inspected to support understanding of how they were used, and to corroborate, illustrate or refute the perspectives expressed in the interviews.

As part of the iterative process of refining and understanding the data, emerging findings were discussed regularly at the multidisciplinary team meetings (HP, JH, SHL, MM, LMcC, BMcK), and presented to the wider team at quarterly steering group meetings. A final feedback meeting, involving the stakeholders (patients, healthcare professionals and managers), invited discussion on the preliminary findings and key issues raised to aid interpretation. The agenda for this meeting is provided in Additional file 5.

## Results

**Fig. 2:**
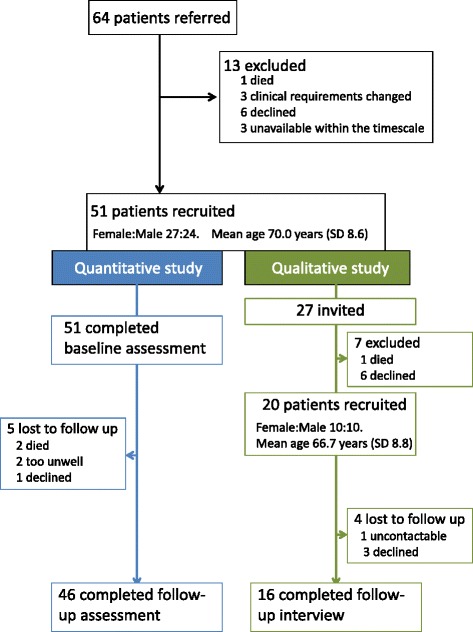
Consort diagram. Flow of patients through the study

Figure 2 illustrates the flow of patients through the study. A total of 51 participants (27 female: mean age 70.0 years (SD = 8.6)) were recruited to the Light-Touch service. Twenty (10 female: mean age 66.7 years (SD = 8.8)) of the 27 who we approached agreed to be interviewed at baseline and 16 participants completed follow up interviews. Demographic details of the participants are given in Table 1. Eight healthcare professionals took part in the focus group (5 specialist respiratory physiotherapists, 2 district nurses, 1 anticipatory care nurse). Six managers participated in individual interviews (4 from Edinburgh and 2 from East and Midlothian).

**Table 1:** Demographics of the patients and professional groups

Patient demographics						
Age group	Number invited		Number recruited		Initial interview	Follow-up interview
	Male	Female	Male	Female		
40–49	0	1	0	1	1	1
50–59	2	2	2	1	3	3
60–69	5	4	5	3	8	6
70–79	3	7	2	4	6	5
80–89	2	1	1	1	2	1
Total	12	15	10	10	20	16
Professional groups						
Group	Invited		Participated		Notes	
Community respiratory team	5		5			
Anticipatory care services	5		3		1 left the service	
					1 did not reply	
Health service managers	9		6		2 had limited knowledge of Light Touch service	
					1 did not reply	

### Quantitative results

#### Patient reported outcome measures

**Fig. 3:**
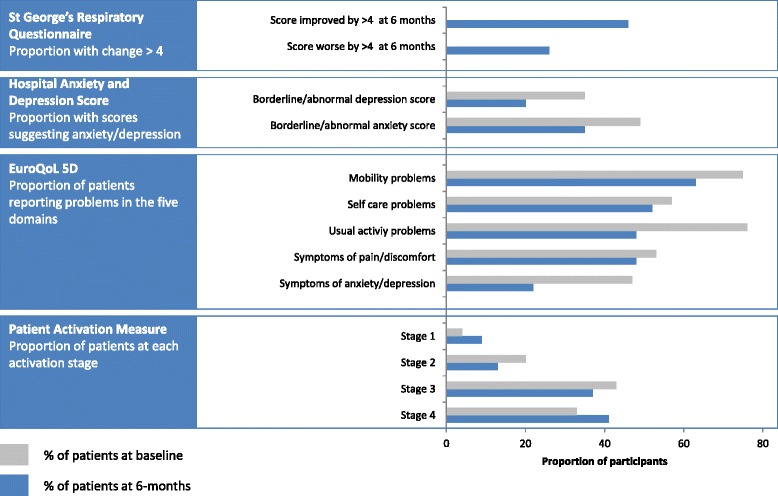
Patient reported outcome measures

The results of the patient reported outcome measures are illustrated in Fig. 3, and numerical results are in Additional file 6. Although we are not presenting a statistical analysis of these before-and-after pilot data, the trend appeared to be towards improved quality-of-life, reduced anxiety and depression, but no change in patient activation over the six-month study. For the SGRQ the individual scores of 21 (46 %) participants improved by ≥ 4 (the minimum clinically important difference [24]); 12 (26 %) deteriorated by ≥4.

### Use of healthcare resources

#### Light-touch service use

**Fig. 4:**
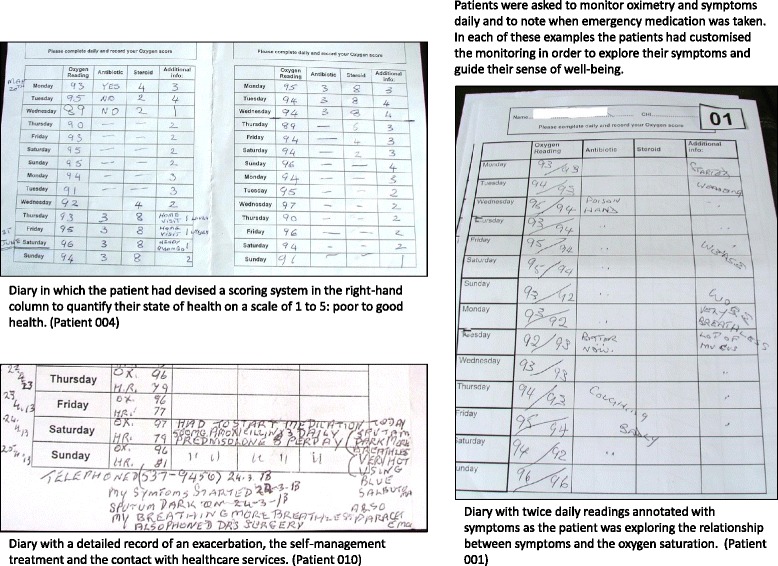
Examples of monitoring diaries

Eighteen (39 %) participants contacted the Light-Touch community-team once or more during the study. Twenty-nine (63 %) participants had recorded pulse oximetry, symptoms and other COPD-related information on diary sheets and had kept this information. Eleven (24 %) participants reported having recorded the information but had not kept or had mislaid it. Six (13 %) had kept no written records. An example of recordings is in Fig. 4.

#### Use of healthcare resources

**Fig. 5:**
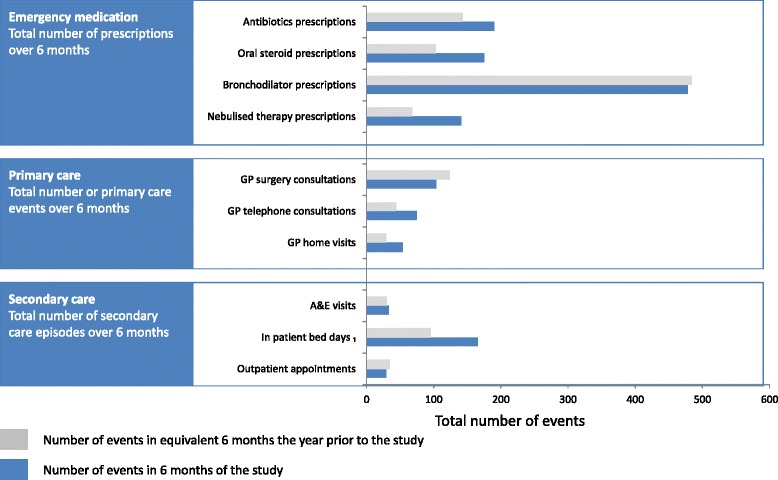
Primary and secondary health care use in the 6-month study compared with same 6 months the previous year

The use of healthcare resources is illustrated in Fig. 5 and detailed in Additional file 6. There was an increase in emergency treatment (oral steroids, antibiotics and nebulised therapy) and a possible shift in mode of general practice consultations from face-to-face to telephone. Use of secondary care (especially duration of admissions) was strongly influenced by a few frequent users of hospital services.

### Qualitative findings

Our data echoed the previously described acceptability of monitoring symptoms and oximetry, [13, 15] and highlighted issues of practical importance to the local service. We here concentrate on data related to the over-arching theme of ‘taking on self-management’ and specifically explore the role of Light-Touch monitoring in enabling patients to self-manage their COPD. The theme is presented from the perspective of the patient and the professionals using the following headings:

1. Taking on self-management: the patients’ perspective. The key themes from the patients’ perspective are: ‘establishing ‘norms”, ‘pulse oximeter as a guide to wellbeing’, ‘enabling control and taking decisions’, ‘taking ownership and reducing reliance on the healthcare professionals’ and ‘stopping routine monitoring’.
2. Relinquishing control: the healthcare professionals’ experiences. The key themes from the professionals’ perspective are: ‘pulse oximeter as a teaching tool’ and ‘relinquishing control: balancing the tensions’.

The data relating to these themes are described below and include illustrative quotes. Additional quotes to support these themes are given in Tables 2 and 3.

**Table 2:** Taking on self-management: the COPD patients’ perspective

Theme	Quote
Establishing the ‘norms’	Exploring the measurements in relation to symptoms
	*“As I told you, it tells you, me the oxygen level. And I take it every day, you can have a look through there, I’ve got all the readings. As you’ll see I’ve got ‘okay’, well the ‘okay’ is that I wasn’t breathless when I took it”.* (Patient 3)
	Involving the family
	*“*[name of wife] *comes over and sticks it on my finger and then gets the sheet and fills it in”.* (Patient 50)
	*“I have a look at mine sometimes. Er, about 92, 93 round about that”.* (Carer 50)
The pulse oximeter as a guide to wellbeing	Confirming state of health
	*“It* [the oximeter] *saves me worrying, getting up tight about my breathing and that. I used to panic, but not since I got that”.* (Patient 26)
	*“So with having that* [the oximeter] *you’re, you’re not having to constantly call your practitioner to come out and make sure that you are okay, so it’s given us the reassurance that if it went too low then we phone* [name of physiotherapist] *and she‘d say ‘phone doctor’…which we did the last time.”* (Carer 19)
	Monitoring recovery
	*“It* [oximeter] *can give you a guide to how you're doing. If you're improving, or not improving, you're taking your antibiotics, you're taking your steroids and it's still going down then you know you're going to get in a bit of trouble”.* (Patient 51)
	Objective evidence
	*“When you do phone for help, the more information you can give them the better it is for the responder, when he comes out. So he understands what’s going on”. (*Patient 51)
Enabling control and taking of decision	Using the oximetry to guide decisions… in conjunction with symptoms
	*“Like I say, I can monitor it, I’m not under 91* [the pulse oximetry reading], *em, my pulse is okay, so just concentrate on breathing, controlling your breathing…and you’ll be okay sort of thing as long as you’re not bringing up phlegm and, or something’s changed. I don’t have to get in touch with anyone; I can self-manage it, you know…”* (Patient 4)
	… or just relying on symptoms
	*“Er, it’s hard to explain, but if it’s like phlegm you’ve got there, it* [chest] *just feels quite tight and sore, and you just want to cough and you’re very breathless and the minute you do anything, it just drains you and makes you more breathless, so that’s probably a bad day”.* (Patient 22)
Taking ownership and reducing reliance on the healthcare professionals	Not needing professional advice…
	*“…I don’t like to be bothering people, eh? I try and manage myself if I can eh? Erm, I mean, but I mean basically as well, I’ve got everything here that the hospital would give me anyway, apart from oxygen. Erm, so I just try and manage myself without erm…I know now how far I can go, and don’t overdo things or you know what I mean?* (Patient 31)
	… or challenging professional advice
	*“She was really worried about me; so worried that she came to the house before she phoned me she actually came to the house for me to see if I was alright, to send me down to the hospital. But I was in North Berwick, so she phoned there and asked me, ‘Do you want to go into hospital?’ ‘No, I don’t need to go.’ ‘But you must! Your sats are so low.’”* (Patient 47)
On-going monitoring (or not)	Nothing to record
	*“No, I can’t be bothered! I’m a lazy devil!* [Laughs] *I suppose if I was ill I would, but…I’ve not got anything to record in it, ken?* (Patient 11)
	No more space in the diary
	*“I’m waiting for them* [community team] *coming but they never seem to come back. Today’s *[reading] *was 90…it’s not here, that’s finished* [indicated end of diary]*”.* (Patient 7)
	Keeping a diary is for the health professionals, not me
	*“The whole idea of this is for the nurses to look after me, if need be, rather than been taken into hospital, eh. Erm, so it’s a long term thing”.* (Patient 31)
	It helps me, even if the service I withdrawn
	*“Well, if it’s taken off me, I’ll buy one for myself, because as I say it gave me mair*[more] *confidence to take control …well to me it’s like a comforter now”.* (Patient 45)

**Table 3:** Relinquishing control: the healthcare professionals’ experience

Theme	Quote
A teaching tool	Oximetry and keeping a diary aid recognition of symptoms of exacerbations
	*“It’s about the education that goes into identifying trends, and trends when they’re well and trends when they’re less well, and if they’re gonna then be able to use the tool to, yes, be able to monitor that, but then also be able to act upon that as well”.* (Physio 3)
	*“When I go in and say to someone ‘Okay, so when you become unwell tell me what you notice.’ What they’re describing to me is an exacerbation that actually is quite set in. They can’t pick up those signs and symptoms earlier on. Um, and that’s something that I didn’t expect the symptom diary to do. I suppose at first I was underestimating how important the symptom diary might be, but for some people it’s actually been the teaching tool”.* (District Nurse 1)
	*“It’s telling them ‘It’s* [oximetry] *not a diagnostic tool.’ I always say to them ‘It’s an add-on to help yourself manage.’”* (District Nurse 3)
	But not for everyone
	*“Er, it’s not a hundred per cent of people, that will want to use technology, and we have to respect that, but we also have to be able to offer, um, in appropriate parts of the patient journey, where it will assist the patients and the staff as well, to work in new ways, to combat, um, long term conditions”. (Manager 1)*
Relinquishing control (Balancing the tensions)	Fear of increased workload: monitoring impact
	*“Um, and we had some just early stats on that, that showed that, you know, that we were increasing our numbers of patients, but the numbers of calls were not necessarily going in the same direction, they were staying quite static, so it meant that we could manage more patients, um, on using the technology but it didn’t increase the workload for the team, as much”.* (Manager 1)
	Some patients need professional reassurance that they were acting correctly….
	*“We have the other, the other side of that where a patient will phone NHS24* [24-h telephone helpline] *and say ‘These are the symptoms I’ve got, my saturations are eighty-nine’ And they* [NHS-24] *will phone an ambulance, and the patient doesn’t want an ambulance…they just want to tell someone ‘Is it okay for me to start my antibiotics with the symptoms I’ve got?’ So they say ‘Right, let’s get you into hospital.’ They don’t want to go to hospital, they just want to inform someone”*
	…. Others delayed seeking advice despite failing to respond
	*“And on occasions you’ll get somebody who phones to say they’ve just finished their course of antibiotics and steroids but they’re no better…I’d say ‘Well, actually I would like to know beforehand that you’re on them so that we can plan ahead, and if you’re not getting better then we need to put something in place’ Get the GP involved, um, do sputum samples, anything like that to try and sort of carry that management forward”.* (Physio 4)

### Taking on self-management: the patients’ perspective

The patients’ perspective on taking on self-management explores the role of oximetry in establishing norms and as a guide to wellbeing, which supported taking treatment decisions and reduced perceived reliance on healthcare professionals.

#### Establishing ‘norms’

The majority of patients used the pulse oximeter to establish their ‘normal’ oxygen saturation levels, using their daily readings to help them understand their health status. See Fig. 4 for examples. Family members or friends sometimes assisted with monitoring and compared their own readings. Some patients described taking additional measurements to explore associations between symptoms and their readings

*“When I first got it I was checking, like you see on that* [symptom diary], *I was checking it like in the evening and then, eh, just to see what the difference was because in the evening, as I say, I’m getting more tired, more tired, more breathless, and I wanted to find out the difference, eh”.* (031: Male/62 years)

In contrast, other patients questioned the benefits to them of the monitoring, and were keeping a diary for others to use.

*“I feel I’m doing it, because I’m thinking it’s going to help them do research, but…I don’t see it doing me any good. It’s not doing me any harm”.*(030: Female/74 years)

#### The pulse oximeter as a guide to wellbeing

Patients described the pulse oximeter as *‘a guide’*, *‘a tool’*, *‘an apparatus’*, ‘*a wee machine’* that gave an immediate indication of their health status. This could *‘prevent panic’* or conversely confirm their suspicion that they were not so well that day. It could obviate the need for a consultation, inform the decision to request professional help, monitor recovery after an exacerbation, or provide objective information to report to a healthcare professional.

*“It* [the oximeter] *is telling you, rather than a doctor saying, you know, with a stethoscope or something like that, that can tell you you’re okay, you’re in the level of…you’re not feeling unwell.”* (019: Male/65 years)

Patients described referring to their management plan, which gave personalised advice based on both symptoms and oximetry.

*“It helps me, it gives me a bit of confidence. ….Any problems, yeah, because you’re actually going by what it says on the front of that* [management plan]*”.* (026: Male/70 year)

There were divergent opinions about whether they had more faith in their oximetry readings, their symptoms, or both:

*“Mm…the reading actually”.* (026: Male/70 year)

*“Well, it’s obviously my symptoms because if they weren’t bad, I wouldn’t be checking* [the oximetry]. *I just use that to check what number I’m at and then decide.”* (011: Female/71 year)

One patient avoided using the oximeter because it reminded her about her COPD and if *“you’re feeling okay, why are you going to keep thinking about it and looking at it every day?”* (030: Female/74 years)

#### Enabling control and taking decisions

Oxygen saturation, in conjunction with symptoms, gave some patients the confidence to make self-management decisions: both day-to-day decisions about living with COPD, and decisions about dealing with exacerbations.

*“…when I first realised I had to be on oxygen, I lost all confidence. Sometimes I wouldnae* [would not] *go out for six or eight weeks. Now I know, and I can take my reading, I can go out any day I want”.* (010: Female/73 years)

*“Well, I wouldn’t start the antibiotics right away. I would just see what I was like within myself, if I was coughing more*, *if my sputum was thicker or jellyish and maybe it was that I would start the antibiotics”.* (045: Female/62 years)

Not everyone agreed with this: some patients were aware of their symptoms and commented that the pulse oximeter did not make a difference.

*“As long as I can breathe and I’m not struggling for breath it’s a good day”.* (014: Female/69 years)

*“I wouldn’t say there’s much difference; I have to be honest with you… it’s not made any difference to my lifestyle or anything”.* (037: Female/80 year)

One patient who had participated in the telemonitoring trial,[13] was able to compare Light-Touch with the fully monitored service, and described the increased autonomy she felt with the Light-Touch service.

*“Erm, they* [the respiratory monitoring team] *were controlling my illness …, whereas now I’m able to keep control of my illness. I know when I’m not well. I’m not having to depend on a box* [the telemonitoring equipment] *to tell me I’m ill. Now I do it myself so I feel a bit more in control now so…”* (027: Female/48 years)

#### Taking ownership and reducing reliance on the healthcare professionals

Some patients described a transition as they developed an understanding of their condition, the significance of their readings and symptoms and were empowered to decide when to self-refer (or not)*.* Despite advice to contact the clinical team if they felt unwell, some patients reported that over time they had become sufficiently confident in their self-management that they did not need – or even felt able to challenge – professional opinion.

*“I just didn’t think there was any need to phone them, because there was nothing else they could have done, …, if I’d have went to the doctor, he’d have just gave me antibiotics, which I started. And if I had to phone the physio, she probably would have told me to just start on the antibiotics anyway, so I’ve done, you know, what I was supposed to do, I think”.* (022: Female/55 years)

#### Stopping routine monitoring

At the 6-month interview, several patients had stopped routine monitoring preferring to check readings as and when the need arose. Some had stopped keeping a record, and the majority of those who continued to keep a diary perceived that they were doing this for the benefit of healthcare professionals rather than themselves.

*“Well, at least that is written down for them* [healthcare professionals], *that’s how my heart beat has been going, and that’s how my oxygen level’s been going. It helps them to understand it, at least, I think so”.* (003: Female/76 years)

### Relinquishing control: the healthcare professionals’ experiences

This theme highlights the professionals’ experience of using the pulse oximeter as a teaching tool, the perceived positive impact on self-management and (sometimes) concern about the reduced contact with some patients who rarely needed professional support.

#### Pulse oximeter as a teaching tool

Healthcare professionals used the diary of symptoms and oxygen saturations to teach patients and their carers to differentiate symptoms, recognise trends and identify early signs of exacerbations.

*“…one of the things that we’ve been using with the* [community] *team is to help support people, especially carers, recognise the symptoms and recognise the difference maybe between anxiety, breathlessness, and actually an exacerbation”. *(District Nurse 1)

One of the managers described oximetry as a tool for supporting self-management by “*bringing the use of technology into the assessment process”.* They perceived that the lack of professional monitoring was *‘great, because it’s their information and they self-manage’.* In the future, however, they hoped to introduce a system that enabled data transmission, so that clinicians (especially in unscheduled care) could access records.

#### Enabling self-management: balancing the tensions

The initial concern was that some patients might be unable to cope without professional monitoring. In the event, few patients contacted the clinical teams for advice during the study.

*“I’ve had quite a few patients say to me, when I’ve got back in contact with them, ‘Oh, I started my antibiotics, I started my steroids because I scored two or more’, but they never phoned in and it’s ‘Oh, I didn’t want to bother you’”.* (Physio 2)

Paradoxically, this resulted in the opposite concern: that there was insufficient contact to enable the Light-Touch professionals to feel that they were providing adequate support and oversight.

*“I think sometimes that’s the problem, where you get a patient who’s maybe self-managing better, they self-manage and they cut out the clinician completely then because they’ve become reasonably good at self-managing. But that patient then can sometimes fall off the radar”.* (Physio 3)

In some cases, the professionals were sufficiently concerned that they had instigated proactive contacts with patients new to the service to check on progress.

*“.. but where we’re different is that we actually instigate a lot of those telephone consultations as well, particularly if somebody has not long started on Light Touch. They may just be chugging along nicely if you like…”* (District Nurse 3)

This was discussed at the feedback meeting, when the professionals reiterated their concern at the reduction in the number of patients contacting the service and described their plans to institute regular telephone reviews to maintain contact.

## Discussion

### Main findings

Light-Touch monitoring proved to be feasible in supporting self-management, and was adapted by patients so that it fitted acceptably into their routines. In our small dataset, we found a trend towards improved quality-of-life and reduced anxiety and depression over the six-month study, and an increase in courses of emergency medication, which reflects the previously reported impact of telemonitoring on increased recognition and treatment of exacerbations [8, 13]. There was a trend towards a shift from face-to-face to telephone consultations in general practice. Only about a third of participants contacted the Light-Touch service at any time during the 6-months.

Oximetry was appreciated by most professionals and patients as a guide to well-being and a tool which provided ‘clinical’ information that gave them the confidence to act in line with their self-management plan. Patients described being able to assume control and reduce reliance on healthcare professionals. This was welcomed by professionals and their managers as evidence of successful promotion of self-management, though there was some concern that the reduced contact might compromise the support and care provided.

### Strengths and limitations of this study

This was a feasibility study and numbers were therefore small, with some attrition mainly due to poor health that would need to be taken into account in planning a fully powered trial. We allowed for seasonal influences by comparing the study period with the same months the previous year, but there may have been other confounding factors that could have affected the outcome. Some patients were offered the intervention following a period of poor health and improvements in outcome may have been due to regression to the mean. All the participants were recruited from one Health Board in Scotland so may not be representative of the wider COPD patient population: specifically none were from an ethnic minority group.

**Fig. 6:**
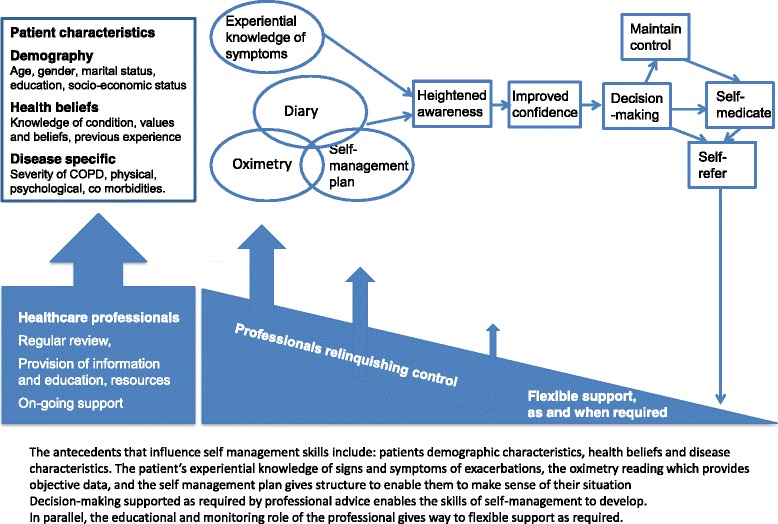
Proposed evolution of self-management

Serial interviews enabled us to capture a process of change in perceptions over time and the evolution of self-management skills (Fig. 6). We were aware that researchers’ attitudes influence design, data collection and analysis of qualitative studies, [25] however, the multidisciplinary team met frequently to discuss emerging findings, and the end-of-project stakeholder meeting at which preliminary conclusions were presented enabled a balanced interpretation.

### Interpretation of findings in relation to previously published work

Telemonitoring is promoted as a strategy for supporting self-management [5–7] though previous studies have suggested that paradoxically it may engender dependence. [15, 26] The unmonitored Light-Touch service appeared to underpin an evolution to supported self-management (illustrated in Fig. 6), consistent with literature which describes empowerment as a process, [27] as well as an outcome [28, 29].

People with COPD use a range of non-specific symptoms/feelings to indicate the onset of an exacerbation,[30] and the opportunity to monitor measurements previously exclusive to healthcare professionals,[31] provided insights into well-being and enabled decision-making despite the lack of a clear predictive value [32].

Healthcare professionals view empowerment as providing knowledge, resources and support, [33] overlooking the key element of relinquishing professional control. [34, 35] We observed an evolution as the educational and monitoring role of the community teams gave way to self-management and ‘as-required’ contact with professionals. This caused some concern amongst the community teams about reduced support and a potentially adverse impact on continuity of care, [36] though they recognised that this may reflect a necessary shift in working practice as services move to economically viable models of supported self-management [37–39].

### Implications for future research, policy and practice

The Light Touch service was a response by the healthcare system to the negative trial of traditional COPD telemonitoring [13]. Our feasibility study enabled us to explore this initiative and learn lessons about how this new approach might work and how the initiative might be refined for further development and evaluation [16]. Bucknall et al. [40] highlighted that only a proportion of people with COPD were able reliably to identify change and effectively self-manage concluding that there was a need for professionals to ‘train’ patients. More subtly, our ‘Light Touch’ data suggest that enabling people to learn experientially combined with ‘supportive withdrawal’ of professional care can allow self-management to evolve. The impact of this on professionals who had previously provided regular monitoring was unexpected and suggests that future iterations of the service need to consider how continuity of care and support can be maintained whilst adopting a Light Touch approach.

## Conclusions

In our feasibility study, oximetry as part of a community-based ‘Light Touch’ service was perceived as promoting understanding of symptoms, and providing the confidence to enable people with severe COPD to assume control of their condition. Although conceived as a successor to telemonitoring, the absence of data transmission and professional monitoring both reduced workload for the professional and seemed to enable self-management to evolve. Further research should seek to evaluate the effectiveness of this approach on clinical outcomes.

## Additional files

Additional file 1:
**Symptom diary.** (PDF 33 kb) Additional file 2:
**Self-management plan.** (PDF 91 kb) Additional file 3:
**Topic guides.** (PDF 584 kb) Additional file 4:
**Coding frameworks.** (PDF 94 kb) Additional file 5:
**Feedback meeting delegates and agenda.** (PDF 48 kb) Additional file 6:
**Quantitative data, a) Patient reported outcome measures before and after Light Touch monitoring, b) Use of healthcare resources in the 6-months of the study compared to the same 6-months in the preceding year.** (PDF 233 kb)

## Abbreviations

COPD: Chronic Obstructive Pulmonary Disease
EQ-5D: EuroQol-5D
HADS: Hospital Anxiety and Depression Scale
PAM: Patient Activation Measure
SGRQ: St George’s Respiratory Questionnaire
