# Supplementary material for: Oximetry-supported self-management for chronic obstructive pulmonary disease: mixed method feasibility pilot project
Source: BMC Health Serv Res. 2015 Oct 26;15:485. doi: 10.1186/s12913-015-1135-2 (PMC4624181; doi:10.1186/s12913-015-1135-2)
Supplement: Additional file 1: — Symptom diary. (PDF 33 kb) [file 12913_2015_1135_MOESM1_ESM.pdf]

NAME: ....

COPD Symptom Diary

Please complete daily and record your Oxygen score

	Oxygen Reading	Antibiotic	Steroid	Additional info:
Monday				
Tuesday				
Wednesday				
Thursday				
Friday				
Saturday				
Sunday				
Monday				
Tuesday				
Wednesday				
Thursday				
Friday				
Saturday				
Sunday				
Monday				
Tuesday				
Wednesday				
Thursday				
Friday				
Saturday				
Sunday				
