# Supplementary material for: Oximetry-supported self-management for chronic obstructive pulmonary disease: mixed method feasibility pilot project
Source: BMC Health Serv Res. 2015 Oct 26;15:485. doi: 10.1186/s12913-015-1135-2 (PMC4624181; doi:10.1186/s12913-015-1135-2)
Supplement: Additional file 2: — Self-management plan. (PDF 91 kb) [file 12913_2015_1135_MOESM2_ESM.pdf]

Name of patient

IF YOU ANSWER YES TO 2 OR MORE OF THE FOLLOWING QUESTIONS THEN PLEASE CALL 0131 XXX XXXX BEFORE 12:00 MONDAY TO SUNDAY.

Please leave a message with your name and contact number and we will get back in touch with you later today.

1. My oxygen saturations are below 92%.	**Yes / No**
2. I have increased wheeze since yesterday	**Yes / No**
3. My sputum has increased in amount and is darker in colour	**Yes / No**
4. I am more breathless than yesterday	**Yes / No**

Additional questions for individual patients:
decreased ex tolerance, increased ankle swelling, increased tiredness

EMERGENCY MEDICATION

Antibiotic – take 1 tab 3 times a day for 7 days
Steroids – take 8 tablets in morning
"Blue" Salbutamol/Bricanyl inhaler – increase to…..
Neb/oxygen/ etc.

OTHER CONTACT NUMBERS

- Community Respiratory Team office for non-urgent calls
  Tel: 0131 XXX XXXX (Monday to Friday – 8:30am to 4:30pm)
- GP practice name and tel number
- NHS 24 08454242424
