# Supplementary material for: Oximetry-supported self-management for chronic obstructive pulmonary disease: mixed method feasibility pilot project
Source: BMC Health Serv Res. 2015 Oct 26;15:485. doi: 10.1186/s12913-015-1135-2 (PMC4624181; doi:10.1186/s12913-015-1135-2)
Supplement: Additional file 3: — Topic guides. (PDF 584 kb) [file 12913_2015_1135_MOESM3_ESM.pdf]

Additional file 3a: Topic guide for patients – 1st interview

1. Introduction

- Introduce myself
- Purpose of the interview
- Inform patient about confidentiality
- Get patient's consent
- Inform patient the interview will take around an hour
- Patient can withdraw from the study at any stage
- Will contact patient for a 2nd interview 6 months later
- Interview will be tape recorded for transcribing purposes
- Ask the name and age of patient
- Occupation

2. Impact about using Light Touch Monitor

- Can you tell me about your experience about the Light Touch monitor?
    - Prompts:
        - Is it helping you?
            - What is the best about it?
            - What is the worst about it?
        - How has it affected you? Was it helpful?
        - Has it made any difference to your life?
            - What is difference? Can you give me an example?
        - If step down from the full kit tele-monitoring, ask about the difference.
        - If on the full kit tele-monitoring, ask whether patient perform the spirometry?
    - Ask briefly:
        - When did you start experiencing problems with your breathing?
        - When were you told that you had COPD?
        - Do you know the cause of your COPD?
            - Do you smoke?

3. Practical issues

- How have you found using it?
- Is it helpful – why? Why not?
- Is it easy to use?
- Do you recommend this for patients with the same condition as you?

- Can you tell me how you normally use it?
    - Prompts:
        - When do you normally take your reading?

        - What do you consider is your normal reading?
        - Are there any changes in the reading during the day/night?
            - How do you feel about these changes?
        - Are there any activities that change the readings?
            - How do you feel about your concern at the moment?
        - Do you have your condition under control?
- Can you tell me if/how Light Touch has helped you managed your health at home?
- What are your expectations of the Light Touch monitor?

4. Impact of Light Touch on daily life (Oximetry and symptom diary)

- Can you tell me how does the Light Touch affect your daily life?
    - Prompts:
        - Do you notice symptoms/listen to what your body tells you?
        - Does keeping a diary helps you take your symptoms into account?
        - Do you sometimes do extra checks in the day?
        - Can you give an example of what a good day would be like for you?
        - Can you give an example of what a bad day is like for you?
        - When you feel poorly, do you make any other checks?
            - What are they?
        - Do you think of your condition all the time?
        - Do you think of the Light Touch monitor all the time?

5. Decision making

- **How do you decide what actions (if any) to take after you take your reading?**
    - Prompts
        - How do you work out what to do?
        - Is there anything else you take into account?
        - Do you have more faith in the reading you get or your symptoms?
        - How do you balance the reading and your symptoms?
        - What do you do if you have a low reading?
            - What actions do you take?
            - When do you take action?
            - Who decides what action you take?
            - Who do you contact if you have any concerns?
            - What symptoms do you normally look out for?
- Can you tell me about your last exacerbation (difficulty in breathing)?

- Can you tell me when you would start your emergency medication (antibiotics and/or steroids)?
- Does your medication helps?
- Do you have other important conditions you have to keep an eye on?
        - Do you monitor them as well?

6. **Use of booklet**
    - Can you tell me your thoughts about the self-help booklet
        - Prompts:
            - How does the booklet help you?
                - Does it help to give you information?
                - Does it help you take action (make decision)?
            - Would you recommend this to someone with the same condition as you?
            - Is there anything in the booklet that needs improvement?
    - Do you have/use any other sources of information/advice?

7. **Family**
    - Can you tell me whether your family are involved in monitoring your condition?
        - Prompts:
            - How do they help you?
            - Do they help you take your reading?
            - Do they remind you to take your reading as you used to?
            - Do they remind you to use it to check whether you are okay?
            - Are your family worried about you?
            - Do you confide to them about your concerns?
    - Can you tell me whether anybody in the family tries out or uses the Light Touch monitor?
        - Prompts:
            - If yes, do you compare your reading with theirs?
            - Are there times you might be concerned?
            - Do you take any actions?

8. **Healthcare professionals**
    - Who are the healthcare professionals who look after you?
        - Prompts:
            - How do they help you?
            - How do you feel about their help?
            - How do you feel about these interactions?
            - Do you know who takes the information you sent to?
    - What happens out of hours?

9. Employment (only if the patient is in employment)

- Can you tell me whether your condition affects your work (if working)?
    - Prompts:
        - Does your employer support you?
        - In what way does your employer support you?
        - How frequent do you use the Light Touch monitor at work?

10. Pulmonary rehabilitation

- Have you been offered any pulmonary rehabilitation programme (or exercise)?
    - Prompts:
        - If no, do you know the reason for this?
        - If yes, how long did you attend the programme?
        - Do you think attending the programme helps you in making decision about your condition?

11. Future plans

- Do you see yourself continuing to use the Light Touch in the next 6 months/a year?
- Do you see monitoring with the Light Touch something you want to do forever?

12. Are there any concerns that you would like to share with me?

- Prompts:
    - Do you anticipate any concerns?

13. Is there anything you would like to add?

Thank the participant

Additional file 3b: Topic guide for professionals – 1st focus group

Focus group protocol: Healthcare professionals
Introduction:

- Welcome and thank you for coming today.
- Introduce self and the co-facilitator
- Housekeeping issues - fire alarm and exits.
- This session will take around an hour.
- You are free to withdraw at any point.
- Please sign the consent form.
- The discussion will be recorded and confidentiality will be maintained.
- The purpose of this focus group is to hear your views/opinions/thoughts about the Light Touch Monitoring service.

Ground rules:

- Please feel free to express your opinion
- We won't be telling people outside this room who said what.
- Everyone has a chance to talk, respect others when they are talking.
- There is no right or wrong answers; even negative comments are useful in gaining an insight about the service
- Is there any question before I start?

Let's start by introducing yourself:

  - Name, professional post, how long you have been in your post.

1. **Implementation of the LTM**
    - How do you select patients for the Light Touch monitoring service?
        - What barriers do you faced in the implementation of the LTM service?
        - How things could have been managed?
    - What facilitate/helped you in the implementation of the LTM service?
        - Information needs of staff/patient/carer
        - Training for staff/patient/carer
    - What instructions/advice do you give the patients regarding:
        - The channel of communications
        - The symptom diary
            - Supply of symptom diary
            - Do you think daily recording is necessary?
            - What to record and for how long?
        - The self management plan booklet – do all patients receive this?
        - The patient's management plan
        - What is your view on the pulse oximeter?
            - Replacement of batteries

2. **Technology and service provision**

- Can you tell me your perceptions and experiences of the technology and Light Touch Monitoring service provision?
    - Is there any changes in the way patients and healthcare professionals relate to each other?
    - Can you provide the service to all who may benefit?
    - What is your view on the pulse oximeter?
- Do you think the LTM helps you to prevent admissions/visits to the Accident & Emergency Department?
    - Is there any increase or reduction in home visits?
    - Is there any difference on patient admission and emergency department visits?
    - Is there any change in the prescribing of medications?
- How do you evaluate the effectiveness of this service?
    - Resources
    - Support for staff/patient/carer
- Do you think receiving a text message from patients on their oxygen saturation level would enhance the service?

3. Perception of the patient's decision making process

- How do you think patients make decisions regarding:
    - Treatment
    - Response to deterioration
    - Need for professional advice
- How do you think the LTM influence this process?
- Do you think the LTM has made a difference on how patients perceive their condition and in self management?
    - How confident are you when the patients take their own oxygen saturation measurement?

4. Healthcare professionals perceptions and experiences of the LTM process

- What is your experience in making decision when a COPD patient using the LTM rings up with low oxygen saturation?
    - Protocol/procedure
    - Confidence in making decisions
    - Steps taken if any concerns
    - How do you feel about the lack of face to face consultation?
- What do you do with the information in patient's symptom diary?
- Do you look at the readings on an exacerbation routinely?
    - If the patients record in scraps of paper
    - If patients did not record
    - Help in decision making
    - Other information to consider
    - Any changes to the symptom diary

- What do you do with the self management plan booklet?
    - Update on self management plan booklet
- How do you handle incoming calls for help?
    - On a rota
    - How do you record?
    - Co-ordination of information

End the session:

- Is there anything else you would like to add?
- Thank the participants

Additional file 3c: Topic guide for managers – 1st interview

Light Touch Monitoring Study
Interviews with managers

Introduction:
- Introduce self
- Purpose of the interview
- Inform that the interview will take an hour
- Participant can withdraw at any point
- Get consent
- Inform confidentiality will be maintained
- Interview will be recorded for transcribing purposes

1. **Participant's details**
    - Ask the participant's name, professional post and how long the participant has been in the post.

2. **Implementation of the LTM service**
    - What is your perception and experience in implementing the Light touch monitoring service?
        - What barriers do you faced in the implementation of the LTM service?
        - How things could have been managed?
    - What facilitate/helped you in the implementation of the LTM service?
        - Information needs staff/patient/carer
        - Training and education for staff/selection of patients
    - What is your experience in setting up the LTM contact facilities?

3. **Technology and service provision**
    - Can you tell me your perception and experiences of the technology and Light Touch monitoring service provision?
    - Is there any changes in the way patients and healthcare professionals relate to each other?
    - How do you evaluate the effectiveness of this service?
    - How do you feel about the lack of face to face consultation?
    - What are your views on the resources?
        - Pulse oximeters
        - Replacement of batteries
        - Self-management plan booklets
        - Symptom diary
        - Support for staff/patients/carers
        - Managing contacts/phone calls from patients/carers

4. Future vision for the Light Touch Monitoring service

- What is your future vision on the Light Touch monitoring service?
  - Can you provide the service to all who may benefit?
  - Do you intend to collect the pulse oximeters back once patients are self-managing with their symptoms?
  - Is there any progress on the Florence service?
  - Is there any plan to refer patients to the LTM service earlier rather than at critical points (presently most patients are referred from hospitals)?
  - What plans are in place to highlight this service to other healthcare professionals?

5. Is there anything you would like to add?

COPD Light Touch Study

Additional file 3d: Topic guide for patients – 2ndinterview

Introduction

- Purpose of the 2ndinterview
- Inform patient about confidentiality
- Get patient's consent
- Inform patient the interview will take up to ½ an hour
- Interview will be tape recorded for transcribing purposes

1. **How is your health since the last time I saw you?**

2. **Impact of LTM on daily life**
    - Can you tell me how you are getting on with the pulse oximeter since then?
    - Has it made any difference to your life?

3. **Practical issues using the pulse oximeter**
    - How have you found using the pulse oximeter?

4. **Decision making**
    - Has using the pulse oximeter helped you manage your health at home?
    - How have you been keeping with the symptom diary?
    - Can you tell me whether you have any exacerbation since the last time we met?
        - If yes: What did you do and who did you contact?
        - Were you admitted to the hospital?

5. **Future plans**
    - How long do you think you will be using this?

6. **Is there anything you would like to add?**

Thank the participant
