# Supplementary material for: Oximetry-supported self-management for chronic obstructive pulmonary disease: mixed method feasibility pilot project
Source: BMC Health Serv Res. 2015 Oct 26;15:485. doi: 10.1186/s12913-015-1135-2 (PMC4624181; doi:10.1186/s12913-015-1135-2)
Supplement: Additional file 4: — Coding frameworks. (PDF 94 kb) [file 12913_2015_1135_MOESM4_ESM.pdf]

Additional file 4a: Coding framework (patients)

Themes	Sub-themes
1. Establishing the 'norm'	• It is part of the routine<br>• Establishing a pattern in the readings
2. The pulse oximeter as a guide to wellbeing in real time	• Interpreting and differentiating the variations of symptoms
3. Gaining control and response to the readings	• Adapting to life with COPD<br>• "Some days I am better, some days I am worse"<br>• Exploring the boundaries
4. Less reliance on the healthcare professionals	• "The healthcare professionals does the exact same thing"<br>• "The readings are for the healthcare professionals to see<br>• Some people don't like control

Additional file 4b: Coding framework (health professionals)

Themes	Sub-themes
1. Selection of patients to self-manage	• Identifying patient's self-management capabilities<br>• Identifying patient's support network
2. Pulse oximeter as a teaching tool	• Interpreting and differentiating the variations of symptoms<br>• Ability to take action to self-medicate
3. Awareness of the 'Light Touch' telemonitoring service	• Service awareness<br>• Being seen as the expert in COPD
4. Reorganisation of care	• Changes in the provision of service
5. Loss of engagement	• Managing the risks/patient safety<br>• Strategies to encourage patients to self refer

Additional file 4c: Coding framework (managers)

Themes	Sub-themes
1. Thinking of technology every part of the patient's pathway	• Preparing staff and patients<br>• New ways to assess and manage long-term conditions in an ageing population
2. Awareness of the 'Light Touch' telemonitoring service	• Service awareness – anticipated work overload<br>• Individualised patient care
3. Reorganisation of care	• Florence service<br>• Telehealthcare<br>• Broad spectrum care<br>• Lack of cost effective care model for NHS Lothian<br>• Logistic of care – Who monitors?
