# Supplementary material for: Oximetry-supported self-management for chronic obstructive pulmonary disease: mixed method feasibility pilot project
Source: BMC Health Serv Res. 2015 Oct 26;15:485. doi: 10.1186/s12913-015-1135-2 (PMC4624181; doi:10.1186/s12913-015-1135-2)
Supplement: Additional file 5: — Feedback meeting delegates and agenda. (PDF 48 kb) [file 12913_2015_1135_MOESM5_ESM.pdf]

Additional file 5.

COPD Light Touch Meeting Schedule (1st April 2014)

Chair: Hilary Pinnock

Delegates: Three patients: and one spouse.

Six members of the community respiratory services (including a manager, physiotherapists, long-term condition nurses, and a student)

Five members of the Light Touch team (SHL, MM, JH, LMcC, BMcK)

Three members of the wider TeleScot team (including SB)

Agenda:

10.00 Welcome – House Keeping

10.15 The COPD Light Touch study initial results – Short presentations (HP, MM, SHL)

11:00 Any questions – Brief opportunity to ask questions about the presentation

11.15 Coffee

11.25 Discussion –facilitated by members of the TeleScot team not directly involved in data collection and analysis. General discussion on agreement/disagreement with the preliminary findings. Specific questions included:

What aspects of Light Touch helped you (your patients) look after your (their) COPD?

Would it make a difference if someone had been monitoring your (their) readings?

How often is routine review required/wanted?

11:50 Summary of discussions. – What happens next? –dissemination.

12.00 Thank you and close
