# Supplementary material for: Oximetry-supported self-management for chronic obstructive pulmonary disease: mixed method feasibility pilot project
Source: BMC Health Serv Res. 2015 Oct 26;15:485. doi: 10.1186/s12913-015-1135-2 (PMC4624181; doi:10.1186/s12913-015-1135-2)
Supplement: Additional file 6: — Quantitative data, a) Patient reported outcome measures before and after Light Touch monitoring, b) Use of healthcare resources in the 6-months of the study compared to the same 6-months in the preceding year. (PDF 233 kb) [file 12913_2015_1135_MOESM6_ESM.pdf]

Additional file 6a: Patient Reported Outcomes

1. St Georges Respiratory Questionnaire (SGRQ)

Scores at baseline and 6-months

	Baseline (n=51)	6 month (n=46)
Median overall score (IQR)	65.64 (53.31-71.99)	57.39 (47.98-65.96)
Median symptoms score (IQR)	82.02 (69.05-90.50)	78.80 (70.30-88.60)
Median activity score (IQR)	70.09 (59.24-85.87)	60.56 (48.62-75.61)
Median impact score (IQR)	51.79 (38.88-67.07)	47.22 (33.96-61.90)

Number (Proportion) with a clinically significant difference (+/- ≥4) at 6-month follow-up

	Male	Female	Overall
Number of participants with improved score (%)	9 (20)	12 (26)	21 (46)
Number of participants with a worse score (%)	7 (15)	5 (11)	12 (26)
Number of participants with no difference (%)	5 (11)	8 (17)	13 (28)

2. Hospital Anxiety and Depression Score (HADS)

Scores at baseline and 6-months

	Baseline (n=51)	6 month (n=46)
Median overall score (IQR)	12.00 (10.00-18.50)	10.00 (7.00-16.25)
Median anxiety score (IQR)	7.00 (4.00-12.00)	6.00 (2.75-8.25)
Median depression score (IQR)	6.00 (3.75-8.00)	4.50 (3.00-6.00)

Number (Proportion) with a borderline/abnormal anxiety score at baseline and 6-months

	Baseline (n=51)	6 month (n=46)
Participants with borderline/abnormal anxiety score (%)	25 (49)	16 (35)
Participants with normal anxiety score (%)	26 (51)	30 (65)
Participants with borderline/abnormal depression score (%)	18 (35)	9 (20)
Participants with normal depression score (%)	33 (65)	37 (80)

3. Patient Activation Measure (PAM)

Scores at baseline and 6-months

	Baseline (n=51)	6 month (n=46)
Median activation score (IQR)	56.40 (56.4-72.85)	56.40 (52.90-73.10)

Number (proportion) at the different stages of activation at baseline and 6-months

	Baseline (n=51)	6 month (n=46)
Number of participants at Stage 1 (%)	2 (4)	4 (9)
Number of participants at Stage 2 (%)	10 (20)	6 (13)
Number of participants at Stage 3 (%)	22 (43)	17 (37)
Number of participants at Stage 4 (%)	17 (33)	19 (41)

4. EuroQol 5D (EQ-5D)

Scores at baseline and 6-months

	Baseline (n=51)	6 month (n=46)
Median overall health state tariff (IQR)	0.59 (0.27-0.74)	0.71 (0.38-0.83)
Mean visual analogue scale score (SD)	52.55 (19.92)	56.22 (18.37)

Number (proportion) with a difference in health state tariffs at baseline and 6-months

	6 month (n=46)
Number reporting a higher health state tariff at six months (%)	28 (61)
Number reporting a lower health state tariff at six months (%)	16 (35)
Number reporting the same health state tariff at six months (%)	2 (4)
Number reporting a higher visual analogue scale score at six months (%)	22(48)
Number reporting a lower visual analogue scale score at six months (%)	16 (35)
Number reporting the same visual analogue scale score at six months (%)	8 (17)

A higher tariff/visual analogue score indicates a better quality of life

Number (proportion) reporting problems in the five dimensions of the EQ-5D at baseline and 6-months.

	Baseline (n=51)	6 month (n=46)
Number reporting problems with mobility (%)	38 (75)	29 (63)
Number reporting problems with self-care (%)	29 (57)	24 (52)
Number reporting problems with usual activities (%)	39 (76)	22 (48)
Number reporting pain/discomfort (%)	27 (53)	22 (48)
Number reporting anxiety/depression (%)	24 (47)	10 (22)

Additional file 6b: Use of healthcare resources

1. Secondary healthcare use

Number of visits in study 6-months and the equivalent 6-months the previous year

	Study 6-months (n=46)	6 month previous year (n=45)
**A&E visits**		
Number (%) of patients visiting A&E at least once	16 (35)	15 (33)
Median number of visits/patient (IQR)	0.00 (0.00-1.00) range 1-6	0.00 (0.00-1.00) range 1-8
**Hospital outpatient appointments**		
Number (%) of patients attending at least once	17 (37%)	18 (40)
Median number of appointments/patient (IQR)	0.00 (0.00-1.00) range 1-4	0.00 (0.00-1.00) range 1-4
**Hospital Inpatient admissions**		
Number (%) of patients with at least one admission	12 (26%)	15 (33)
Median length of hospital stay in bed days per patient (IQR)	0.00 (0.00-2.00) [a] range 1-38	0.00 (0.00-1.50) [b] range 1-31

[a] 5(42%) of the 12 inpatients accounted for 74% of the days in hospital
[b] 4 (27%) of the 15 inpatients accounted for 70% of the days in hospital

2. Primary healthcare use

Number of GP consultations in study 6-months and the equivalent 6-months the previous year

	Study 6-months (n=46)	6 month previous year (n=45)
**GP surgery consultations**		
Number (%) of patients with at least one GP surgery consultation	28 (61)	34 (78)
Median number of GP surgery consultations/patient (IQR)	1.00 (0.00-2.00) range 1-8	0.00 (0.00-1.00) range 1-5
**GP home visits**		
Number (%) of patients with at least one GP home visit	26 (57)	13 (26)
Median number of GP home visitds/patient (IQR)	1.00 (0.00-4.50) range 1-6	2.00 (0.50-4.50) range 1-5
**GP telephone consultations**		
Number (%) of patients with at least one GP telephone consultation	27 (59)	16 (36)
Median number of GP telephone consultations/ patient (IQR)	1.00 (0.00-2.00) range 1-10	0.00 (0.00-1.50) range 1-8

3. Medication prescribed

Number and proportion of patients receiving prescriptions and the median (IQR) prescriptions/patient in the study 6-months and the equivalent 6-months the previous year,

	Study 6-months (n=46)	6 month previous year (n=45)
**Antibiotics**		
Number (%) of patients prescribed antibiotics at least once	43 (93)	32 (71)
Median number of antibiotic prescriptions per patient (IQR)	3.00 (2.00-6.50) range 1-11	2.00 (0.00-4.50) range 1-16
**Oral prednisolone**		
Number (%) of patients prescribed oral prednisolone (steroid) at least once	39 (85)	29 (64)
Median number of oral prednisolone prescriptions per patient (IQR)	3.0 (1.00-5.50) range 1-14	2.00 (0.00-3.00) range 1-12
**Nebulised bronchodilators**		
Number (%) of patients prescribed nebulised therapy at least once	24 (52)	17 (38)
Median number of nebulised therapy prescriptions per patient (IQR)	1.00 (0.00-4.00) range 1-17	0.00 (0.00-2.00) range 1-8
**Bronchodilator inhalers**		
Number (%) of patients prescribed inhaled bronchodilators at least once	39 (85)	29 (64)
Median number of inhaled bronchodilator prescriptions per patient (IQR)	3.0 (1.00-5.50) range 1-14	2.00 (0.00-3.00) range 1-12
